# Yellow Jack—How Yellow Fever Ravaged America and Walter Reed Discovered Its Deadly Secrets

**DOI:** 10.3201/eid1110.050958

**Published:** 2005-10

**Authors:** Stanton Cope

**Affiliations:** * Naval Institute for Dental and Biomedical Research, Great Lakes, Illinois, USA

**Keywords:** yellow fever, Walter Reed, Cuba, book review

Yellow Jack is a compelling and thorough narrative of one of the most interesting chapters in medical history. The book, which is highly readable even to those who may think they know this story, is based on a series of articles written by one of the authors, John R. Pierce, for Stripe, a publication of the Walter Reed Army Medical Center. Dr Pierce was a colonel in the Army Medical Corps and recently retired after 30 years of active duty service.

The first 5 chapters describe the introduction of yellow fever in North America before 1900. Of particular interest is the chapter detailing events of the 1793 outbreak in Philadelphia, and the efforts of Benjamin Rush to treat patients and determine the specific cause. Chapter 6 compares the roles played by Carlos Juan Finlay and George Miller Sternberg before and during the work of the US Army Yellow Fever Board. Dr Finlay was a Cuban physician who had theorized that mosquitoes transmitted the yellow fever virus, while Sternberg, a US Army physician, claimed to have discovered a bacterium that was the etiologic agent of yellow fever.

Most of the remaining 10 chapters primarily discuss the work of the US Army Yellow Fever Board, led by Major Walter Reed. Yellow fever had ravaged North America for >200 years, bringing death and economic ruin to several major cities. Major Reed designed a series of simple experiments, using human volunteers, which clearly showed that yellow fever was transmitted only by the bite of infected mosquitoes and not by contaminated items or "poison air." Although the story is familiar to some, the authors present an exciting narrative with details not available elsewhere in the literature. The periodic use of quotations from letters and original sources is most welcome.

The book includes a useful Notes section and an extensive bibliography. Additionally, 12 pages of photos and illustrations are provided, some of which are not as clear as one might wish. Overall, however, this book is a valuable addition to the literature on medical history. It will have broad appeal to scientists and nonscientists alike because of the nature of the story, the magnitude of the problem that was solved, and the easy-to-read writing style of the authors. I recommend it highly.

